# A novel case of Staphylococcus pseudintermedius aortitis

**DOI:** 10.1099/acmi.0.000912.v3

**Published:** 2025-03-28

**Authors:** Dione Jones, Matthew Lim, Maxwell Olenski

**Affiliations:** 1 The University of Adelaide, Adelaide, South Australia, Australia, 5005; 2 Alice Springs Hospital, The Gap, Alice Springs, Northern Territory, Australia, 0870; 3 Royal Adelaide Hospital, Adelaide, South Australia, Australia, 5000; 4 Royal Darwin Hospital, Tiwi, Northern Territory, Australia, 0810

**Keywords:** aortitis, cardiovascular infection, *Staphylococcus pseudintermedius*, zoonotic infection

## Abstract

*Staphylococcus pseudintermedius* is a common commensal and opportunistic canine pathogen with emerging pathogenicity in humans. We describe the first case of invasive *S. pseudintermedius* infection causing aortitis and mycotic aneurysm in an 83-year-old patient, treated successfully with flucloxacillin. This case highlights the potential for *S. pseudintermedius* to cause serious endovascular infection in humans.

## Data Summary

All data associated with this work is reported within the article.

## Introduction

Aortitis is a rare but serious condition characterized by inflammation of the aorta, which can be life-threatening if left untreated. The large vessel vasculitides [Takayasu arteritis and giant cell arteritis (GCA)] account for the majority of cases; however, infectious aortitis is well described, albeit less commonly [1]. Infection usually occurs due to haematological spread or transfer from contiguous structures [2] and tends to affect older individuals with pre-existing aortic pathology such as aneurysm or atherosclerosis [1]. *Staphylococcus aureus*, *Salmonella* spp. and *Streptococcus* spp. are the most implicated pathogens [2].

The clinical presentation of aortitis is nonspecific and varies widely depending on the location of inflammation (e.g. ascending thoracic vs abdominal aorta), the arterial branches involved and the underlying cause. Patients may present with constitutional symptoms (fever, weight loss and lethargy), back and/or abdominal pain, features of localized ischaemia or complications such as dissection or thrombosis with peripheral embolization [1]. Imaging modalities such as computed tomography (CT) angiography or magnetic resonance (MR) angiography are usually required for establishing the diagnosis. Depending on whether the underlying aetiology is rheumatological or infectious, management involves either immunosuppressive or antimicrobial therapy, and often consultation with a vascular surgeon [1]. As such, determining the underlying cause of aortitis is crucial.

*Staphylococcus pseudintermedius* is a Gram-positive, coagulase-positive bacteria with similar virulence properties to *S. aureus* [3]. It is a common commensal pathogen well-known for causing skin and soft tissue infections (SSTIs) in dogs [4], yet it is rarely implicated in human disease. Recently, however, *S. pseudintermedius* has been increasingly recognized for its emerging pathogenicity in humans. Case reports have described human *S. pseudintermedius* skin/soft tissue and respiratory tract infections, and less commonly invasive infections such as bacteraemia or endocarditis [5]. We report the first known case of aortitis associated with invasive *S. pseudintermedius* infection in an 83-year-old patient.

## Clinical record

**Fig. 1.**
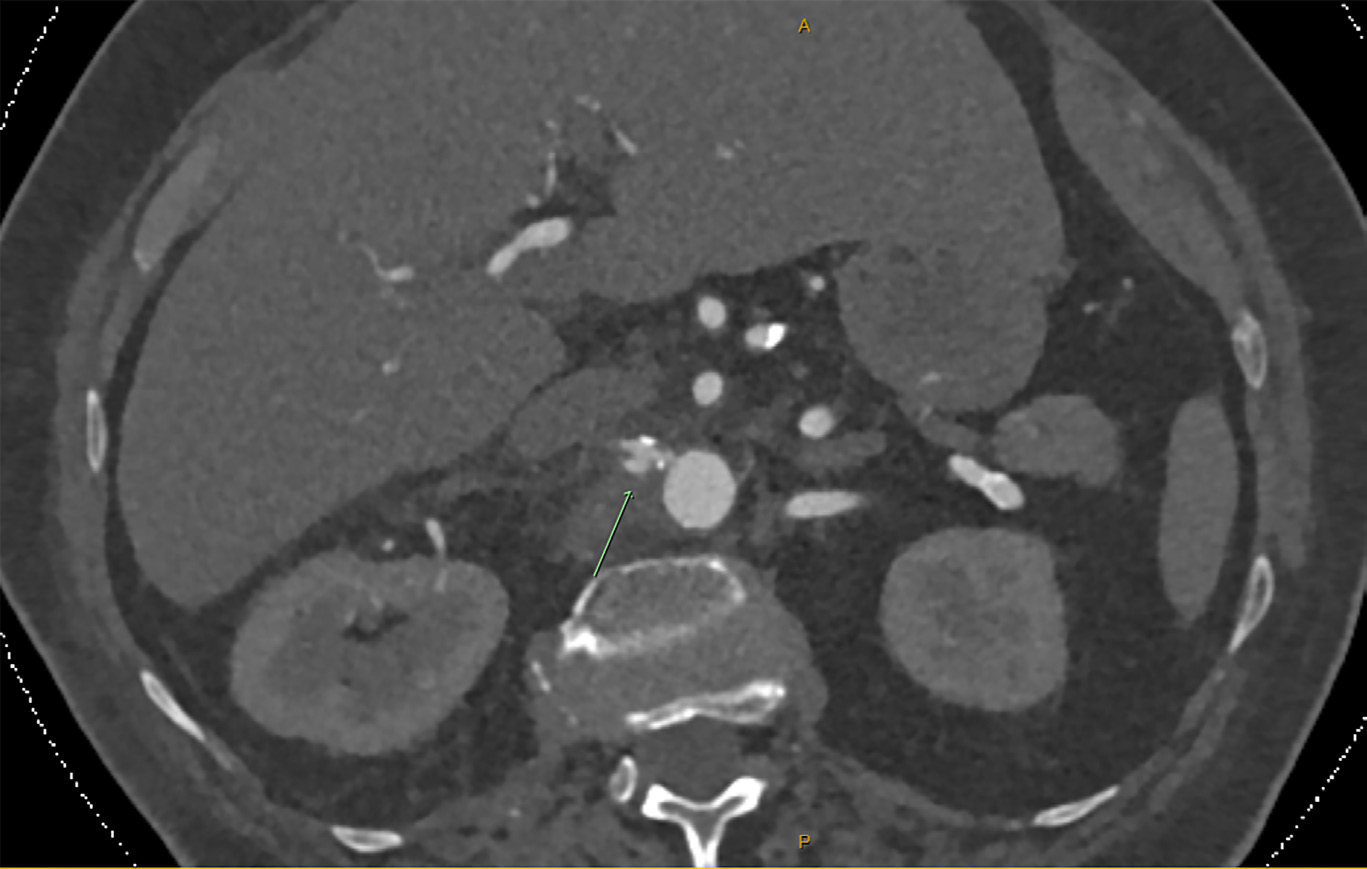
Axial slice from abdominal CT angiogram demonstrating circumferential periaortic fat stranding involving the right renal artery (arrow), indicative of upper abdominal aortitis.

An 83-year-old Caucasian female was admitted with a 1-week history of progressive right-sided flank pain. Her past medical history included asthma, chronic kidney disease, atrial fibrillation and type 2 diabetes mellitus. On initial assessment, she was haemodynamically stable, looked well and was pain-free after simple analgesia. However, contrast CT imaging (confirmed with subsequent CT angiography) revealed focal upper abdominal aortitis extending into both renal arteries with a small, localized dissection, concerning for mycotic aneurysm (Fig. 1).

She was initially treated with empiric intravenous (IV) piperacillin-tazobactam and vancomycin and discussed with the nearest vascular surgery department. The patient was offered percutaneous stent insertion but declined interhospital transfer in favour of conservative management. Both initial blood culture bottles collected upon arrival flagged positive for Gram-positive cocci in clusters, which were *Staphylococcus* latex, DNase and tube coagulase positive. The organism was identified as *S. pseudintermedius* using VITEK (bioMérieux) after 48 hours of incubation. In the context of empiric broad-spectrum antibiotics, subsequent cultures yielded no growth. Sensitivity results revealed resistance to penicillin with susceptibility to flucloxacillin, vancomycin, trimethoprim/sulphamethoxazole and cefazolin. In the absence of evidence of an alternative cause for aortitis (including contiguous vessel involvement, negative syphilis serology and no history or evidence of rheumatological disease), this was favoured as the likely cause for her presentation. Piperacillin-tazobactam and vancomycin were rationalized to IV flucloxacillin monotherapy with a planned duration of 6 weeks to be delivered via outpatient parenteral antibiotic therapy (OPAT). She was discharged following clinical improvement, with weekly monitoring of renal and liver function via OPAT. Subsequently, she had multiple readmissions for fluid overload and acute-on-chronic renal impairment, which was felt to be secondary to the mycotic renal artery aneurysm. This was treated with cautious diuresis and renal adjustment of antimicrobial therapy and was met with gradual improvement in her renal function.

## Discussion

### Aortitis

Aortitis is a rare condition characterized by inflammation of the aorta, which can be caused by infectious and non-infectious aetiologies and can lead to life-threatening complications such as dissection, aneurysm or rupture. The large vessel vasculitides – Takayasu arteritis and GCA – are the most common non-infectious causes, both of which are characterized by granulomatous inflammation of the aorta or its major branches and can usually be distinguished by their age of onset. Takayasu arteritis more commonly affects Asian females, with disease onset typically prior to 40 years of age, whereas GCA more commonly affects Caucasians, with disease onset almost exclusively after age 50 years [6]. Non-infectious aortitis can also occur in the setting of chronic inflammatory or rheumatic disease such as rheumatoid arthritis, systemic lupus erythematosus, sarcoidosis and IgG4-related disease [2].

Infectious aortitis is much less common and more likely to occur in individuals with increased age and pre-existing aortic pathology, such as aneurysm or atherosclerosis [1]. Cases can be associated with bacteraemia (>50%) and/or mycotic aneurysm, which carries a high risk of rupture and mortality if left untreated [1]. The most commonly isolated organisms include *S. aureus*, *Salmonella* spp. and *Streptococcus* spp., although other organisms including mycobacteria, *Treponema pallidum* subspecies *pallidum* and fungi have been implicated [2]. Infectious aortitis has a much higher in-hospital mortality rate than non-infectious aortitis, between 21 and 44% usually due to sepsis [2].

The clinical presentation of aortitis is variable depending on the underlying cause, the distribution of vascular inflammation and the presence or lack of coexisting branching vessel arteritis. Constitutional symptoms (fever, fatigue and weight loss) are common regardless of aetiology, whilst later localized ischaemic symptoms can arise due to progressive narrowing of branching vessels. GCA has a characteristic predilection for the cranial branches of the aortic arch, which can manifest as headache, jaw claudication and, in severe cases, vision loss. The distribution of vascular pathology in Takayasu arteritis is more variable, although it commonly begins in the left subclavian artery [6]. Depending on the vessels affected, symptoms can include arthralgia, limb claudication, pulse or blood pressure discrepancies, hypertension, carotid tenderness, angina, gastrointestinal upset and respiratory or neurological symptoms [6]. The presentation of infectious aortitis is often nonspecific and can mimic non-infectious aortitis [2]. Infected aortic or iliac aneurysms may present with abdominal or back pain, although some cases may only present as pyrexia of unknown origin [7].

Diagnosis of aortitis is usually established radiographically with CT angiography or MR angiography. Differentiation between non-infective and infective causes can be difficult as their presentations can be similar; however, the distinction is critical as the management varies greatly. The former requires immunosuppression, whilst the latter requires prolonged antimicrobial therapy and often surgery [2]. Blood cultures should be collected early in the workup of aortitis to increase the likelihood of isolating a causative pathogen in the case of infectious aortitis, to both aid diagnosis and guide antimicrobial therapy.

### S. pseudintermedius

*S. pseudintermedius* is a Gram-positive, coagulase-positive bacterium possessing similar virulence properties to *S. aureus*. These include staphylococcal protein A, pathogenic enzymes (coagulase, DNase and lipase), secreted toxins (beta-haemolysin and Panton-Valentine leucocidin), superantigens, enterotoxin and the ability to form biofilm [3]. It is a common commensal bacterium and opportunistic pathogen in dogs, known to cause canine skin and ear infections [4]. Whilst it has been reported to colonize up to 90% of healthy dogs, isolates have been identified in a wide range of hosts including cats, horses, foxes, birds and other species [8]. Human colonization rates are not well known.

*S. pseudintermedius* is considered part of the Staphylococcus Intermedius Group owing to its morphological similarities to *S. intermedius*, another coagulase-positive *Staphylococcus*. Until recent years, many automated and phenotypic systems have been unable to differentiate *S. pseudintermedius* from *S. aureus* or *S. intermedius*, resulting in previous misidentifications of *S. pseudintermedius* as the above two species [3]. It was not until 2005 that *S. pseudintermedius* was formally recognized as a novel species [9].

In recent years, *S. pseudintermedius* has been increasingly recognized as a zoonotic organism with pathogenic potential in humans. The majority of reported human infections have been SSTIs in patients with confirmed exposure to dogs; however, respiratory tract infections (sinusitis, otitis and pneumonia) and invasive infections including bacteraemia, endocarditis, prosthetic joint and cardiac device-associated infections have also been reported [5]. To our knowledge, this is the first reported case of focal aortitis in the setting of invasive *S. pseudintermedius* infection, the first case of human infection caused by *S. pseudintermedius* in Australia, and the ninth case of human *S. pseudintermedius* bacteraemia. Table 1 outlines the clinical infections caused by *S. pseudintermedius* that have been reported in humans. Risk factors for human *S. pseudintermedius* infection are not well described, although common patient factors include a history of dog exposure, advanced age and immunocompromise [5]. Notably, the patient in this case did not have any known history of recent dog exposure; however, they did report exposure to a pet bird. Owing to resource limitation, the carrier status of the bird – and its significance in this case – remains unknown as it was not referred for veterinary testing.

**Table 1.** Clinical diseases caused by *S. pseudintermedius* in humans (adapted from [3,8, 12)

Disease	Authors	References
SSTI	Lozano *et al*. 2017Somayaji *et al*. 2016Robb *et al*. 2017Chandak *et al*. 2019Kmieciak and Szewczyk 2018Wegener *et al*. 2022	[13][5][14][15][16][17]
Wound infection	Sawhney *et al*. 2023Wegener *et al*. 2021Pompilio *et al*. 2015Savini *et al.* 2013Starlander *et al*. 2014Bobbitt *et al*. 2022	[18][19][20][21][22][23]
Post-operative wound infection	Blondeau *et al.* 2020LaRocca *et al*. 2023Ahmed *et al*. 2020	[24][25][26]
Dog bite wound	Borjesson *et al*. 2014Moodley *et al*. 2013	[27][28]
Pneumonia	Small *et al*. 2021Asleh *et al*. 2022Olivo Freites *et al*. 2022Laurens *et al*. 2012	[29][30][31][32]
Respiratory tract infection (unspecified)	Sawhney *et al*. 2023	[18]
Rhinosinusitis	Stegmann *et al*. 2010Kuan *et al*. 2016	[33][34]
Otitis	Somayaji *et al*. 2016Raheema 2021Kmieciak and Szewczyk 2018Wegener *et al*. 2021	[5][35][16][19]
Ophthalmia	Kmieciak and Szewczyk 2018	[16]
Cheilitis	Wang *et al*. 2023	[36]
UTI	Blondeau *et al*. 2022Wegener *et al*. 2021	[37][19]
Rectum infection	Wegener *et al*. 2021	[19]
Septic arthritis	Bowen *et al*. 2021Wegener *et al*. 2021	[38][19]
Osteomyelitis	Viau *et al*. 2015Gagetti *et al*. 2020	[39][40]
Prosthetic joint infection	Somayaji *et al*. 2016	[5]
Metalwork infection of the arm	Blondeau *et al*. 2021	[41]
Spinal infection	Darlow *et al*. 2017	[42]
Peritoneal dialysis-associated peritonitis	Dahbour *et al*. 2020	[43]
Implanted endovascular port infection	Nomoto *et al*. 2020Kitagawa *et al*. 2021	[44][45]
Bacteraemia	Chuang *et al*. 2010Somayaji *et al*. 2016Diaz *et al*. 2019Blondeau *et al*. 2020Östholm Balkhed *et al*. 2023Subedi *et al*. 2021Small *et al*. 2021Asleh *et al*. 2022	[46][5][47][24][48][49][29][30]
Cardiac device-associated endocarditis	Van Hoovels *et al*. 2006Riegel *et al.* 2010	[50][51]
Aortitis with mycotic aneurysm and bacteraemia	Jones *et al*. 2025	This study

UTIurinary tract infection

Concerningly, there are reports of increasing antimicrobial resistance in the veterinary sector, including beta-lactam, methicillin-resistant *S. pseudintermedius* (MRSP) and multidrug resistance. MRSP has been considered the veterinary equivalent of hospital and community-acquired methicillin-resistant *S. aureus* (MRSA), yet MRSP is typically resistant to more antibiotic classes than MRSA [10]. The prevalence of MRSP amongst *S. pseudintermedius* isolates in Australian dogs is ~12.7%[10]. Whilst MRSP infection in humans is rare, the increasing prevalence of MRSP in animals combined with the rising incidence of human *S. pseudintermedius* poses a significant public health concern. Accurate laboratory identification of *S. pseudintermedius* remains critical to monitoring resistance patterns, limiting the spread of disease and guiding appropriate antimicrobial therapy. This case highlights the importance of transdisciplinary communication between the veterinary and human health sectors with a One Health approach, to support effective infection control measures and reduce the impact of *S. pseudintermedius* in both humans and animals.

Management guidelines of canine *S. pseudintermedius* infections are described; however, guidelines for managing *S. pseudintermedius* infection in humans are lacking [10]. Given similarities between *S. pseudintermedius* and *S. aureus*, the initial antimicrobial management mirrored that of *S. aureus* bacteraemia and was later guided by sensitivity results. Due to advanced age, frailty and patient preference, a non-surgical approach was followed, and the patient was treated with IV flucloxacillin for 6 weeks with dosing in line with current antimicrobial guidelines for endovascular infection [11].

## Conclusion

We report the first case of aortitis in the setting of invasive *S. pseudintermedius* infection in a human and the first case of invasive human *S. pseudintermedius* infection in Australia. *S. pseudintermedius* has similar virulence to *S. aureus* and ought to be treated with a similar antimicrobial approach to that of invasive *S. aureus* endovascular infection. There is a high prevalence of antimicrobial-resistant *S. pseudintermedius* amongst animals, including methicillin resistance and multidrug resistance. This case underscores the importance of accurate laboratory identification of *S. pseudintermedius*, the need for heightened physician awareness of this emerging pathogen and the ongoing value of a One Health approach in monitoring the spread of antimicrobial resistance.

## Abbreviations

CT: computed tomography
GCA: giant cell arteritis
IV: intravenous
MR: magnetic resonance
MRSA: methicillin-resistant *Staphylococcus aureus*
MRSP: methicillin-resistant *Staphylococcus pseudintermedius*
OPAT: outpatient parenteral antibiotic therapy
SSTIs: skin and soft tissue infections

## References

[1] Gornik HL, Creager MA (2008). Aortitis. Circulation.

[2] Pugh D, Grayson P, Basu N, Dhaun N (2021). Aortitis: recent advances, current concepts and future possibilities. Heart.

[3] Bhooshan S, Negi V, Khatri PK (2020). *Staphylococcus pseudintermedius:* an undocumented, emerging pathogen in humans. GMS Hyg Infect Control.

[4] Carroll KC, Burnham C-AD, Westblade LF (2021). From canines to humans: clinical importance of *Staphylococcus pseudintermedius*. PLOS Pathog.

[5] Somayaji R, Priyantha MA, Rubin JE, Church D (2016). Human infections due to *Staphylococcus pseudintermedius*, an emerging zoonosis of canine origin: report of 24 cases. Diagn Microbiol Infect Dis.

[6] Merkel PA, Warrington KJ, Seo P, Connor RF UpToDate.

[7] Spelman D, Eidt JF, Mills JL, Sexton DJ, Baron EL, Connor RF UpToDate.

[8] Roberts E, Nuttall TJ, Gkekas G, Mellanby RJ, Fitzgerald JR (2024). Not just in man’s best friend: a review of *Staphylococcus pseudintermedius* host range and human zoonosis. Res Vet Sci.

[9] Lynch SA, Helbig KJ (2021). The complex diseases of *Staphylococcus pseudintermedius* in canines: where to next?. Vet Sci.

[10] Australian infectious diseases advisory panel (AIDAP) (2022). Antibiotic Prescribing Detailed Guidelines, 2nd edition.

[11] Lam JC, Stokes W (2023). The golden grapes of wrath - *Staphylococcus aureus* bacteremia: a clinical review. Am J Med.

[12] Moses IB, Santos FF, Gales AC (2023). Human colonization and infection by *Staphylococcus pseudintermedius*: an emerging and underestimated zoonotic pathogen. Microorganisms.

[13] Lozano C, Rezusta A, Ferrer I, Pérez-Laguna V, Zarazaga M (2017). *Staphylococcus pseudintermedius* human infection cases in Spain: dog-to-human transmission. Vector Borne Zoonotic Dis.

[14] Robb AR, Wright ED, Foster AME, Walker R, Malone C (2017). Skin infection caused by a novel strain of *Staphylococcus pseudintermedius* in a Siberian husky dog owner. JMM Case Rep.

[15] Chandak R, Dadu A, Sharma N (2019). *Staphylococcus pseudintermedius*: an emerging cause of pyoderma in pet dog owner. IJCR.

[16] Kmieciak W, Szewczyk EM (2018). Are zoonotic *Staphylococcus pseudintermedius* strains a growing threat for humans?. Folia Microbiol.

[17] Wegener A, Duim B, van der Graaf-van Bloois L, Zomer AL, Visser CE (2022). Within-household transmission and bacterial diversity of *Staphylococcus pseudintermedius*. Pathogens.

[18] Sawhney SS, Vargas RC, Wallace MA, Muenks CE, Lubbers BV (2023). Diagnostic and commensal *Staphylococcus pseudintermedius* genomes reveal niche adaptation through parallel selection of defense mechanisms. Nat Commun.

[19] Wegener A, Broens EM, van der Graaf-van Bloois L, Zomer AL, Visser CE (2021). Absence of host-specific genes in canine and human *Staphylococcus pseudintermedius* as inferred from comparative genomics. Antibiotics.

[20] Pompilio A, De Nicola S, Crocetta V, Guarnieri S, Savini V (2015). New insights in *Staphylococcus pseudintermedius* pathogenicity: antibiotic-resistant biofilm formation by a human wound-associated strain. BMC Microbiol.

[21] Savini V, Barbarini D, Polakowska K, Gherardi G, Białecka A (2013). Methicillin-resistant *Staphylococcus pseudintermedius* infection in a bone marrow transplant recipient. J Clin Microbiol.

[22] Starlander G, Börjesson S, Grönlund-Andersson U, Tellgren-Roth C, Melhus A (2014). Cluster of infections caused by methicillin-resistant *Staphylococcus pseudintermedius* in humans in a tertiary hospital. J Clin Microbiol.

[23] Bobbitt K, Winder ML, Kvas SP (2022). Case report of a diabetic foot infection caused by *Staphylococcus pseudintermedius*, a zoonotic pathogen of canine origin. J Am Podiatr Med Assoc.

[24] Blondeau LD, Rubin JE, Deneer H, Kanthan R, Morrison B (2020). Persistent infection with *Staphylococcus pseudintermedius* in an adult oncology patient with transmission from a family dog. *J Chemother*.

[25] LaRocca MC, Dermarkarian CR, Ediriwickrema LS, Tao JP (2023). *Staphylococcus pseudintermedius* of a semicircular facial flap with concomitant COVID-19 infection. Can J Ophthalmol.

[26] Ahmed AM, Patel PD, Sadiq-Ali S, Lewis PO (2020). *Staphylococcus pseudintermedius* as an emerging coagulase-positive infection in humans. Infect Dis Clin Pract.

[27] Börjesson S, Gómez-Sanz E, Ekström K, Torres C, Grönlund U (2015). *Staphylococcus pseudintermedius* can be misdiagnosed as *Staphylococcus aureus* in humans with dog bite wounds. Eur J Clin Microbiol Infect Dis.

[28] Moodley A, Riley MC, Kania SA, Guardabassi L (2013). Genome sequence of *Staphylococcus pseudintermedius* strain E140, an ST71 european-associated methicillin-resistant isolate. Genome Announc.

[29] Small C, Beatty N, El Helou G (2021). *Staphylococcus pseudintermedius* bacteremia in a lung transplant recipient exposed to domestic pets. Cureus.

[30] Asleh M, Feinstein Y, Lazar I, Rokney A, Baum M (2022). Severe pneumonia caused by methicillin-resistant *Staphylococcus pseudintermedius* in an oncology patient: case report and literature review. Microb Drug Resist.

[31] Olivo Freites C, Sy H, Miguez P, Salonia J (2022). Uncommon pathogens in an immunocompetent host: respiratory isolation of *Cunninghamella bertholletiae, Aspergillus niger, Staphylococcus pseudintermedius* and adenovirus in a patient with necrotising pneumonia. BMJ Case Rep.

[32] Laurens C, Marouzé N, Jean-Pierre H (2012). *Staphylococcus pseudintermedius* et *Pasteurella dagmatics* associés dans un cas de pneumonie communautaire [*Staphylococcus pseudintermedius* and *Pasteurella dagmatics* associated in a case of community- acquired pneumonia]. Med Mal Infect.

[33] Stegmann R, Burnens A, Maranta CA, Perreten V (2010). Human infection associated with methicillin-resistant *Staphylococcus pseudintermedius* ST71. J Antimicrob Chemother.

[34] Kuan EC, Yoon AJ, Vijayan T, Humphries RM, Suh JD (2016). Canine *Staphylococcus pseudintermedius* sinonasal infection in human hosts. Int Forum Allergy Rhinol.

[35] Raheema R (2021). Isolation of methicillin-resistant *staphylococcus pseudintermedius* strains from human otitis cases in wasit province, iraq. Sys Rev Pharm.

[36] Wang S, Nurxat N, Wei M, Wu Y, Wang Q (2023). Cheilitis in an atopic dermatitis patient associated with co-infection of *Staphylococcus pseudintermedius* and *Staphylococcus aureus*. BMC Microbiol.

[37] Blondeau LD, Deutscher M, Rubin JE, Deneer H, Kanthan R (2022). Urinary tract infection in a human male patient with *Staphylococcus pseudintermedius* transmission from the family dog. J Chemother.

[38] Bowen S, DeMarco A, Villasis L, Barsi J, Handel AS (2021). One affectionate puppy: a case of septic arthritis due to *Staphylococcus Pseudintermedius*. Pediatr Infect Dis J.

[39] Viau R, Hujer A, Hujer K, Bonomo R, Jump R (2015). Are *Staphylococcus intermedius* infections in humans cases of mistaken identity? a case series and literature review. Open Forum Infect Dis.

[40] Gagetti P, Errecalde L, Wattam AR, De Belder D, Ojeda Saavedra M (2020). Characterization of the first *mec*A-positive multidrug-resistant *Staphylococcus pseudintermedius* isolated from an argentinian Patient. Microb Drug Resist.

[41] Blondeau LD, Sanche S, Sauder DJ, Deneer H, Kanthan R (2021). Recovery of borderline oxacillin-resistant *Staphylococcus pseudintermedius* (BORSP) from bone and soft tissue of a rheumatoid arthritis patient with severe osteoporosis: transmission from the family dog. *J Chemother*.

[42] Darlow CA, Paidakakos N, Sikander M, Atkins B (2017). A spinal infection with *Staphylococcus pseudintermedius*. *BMJ Case Reports*.

[43] Dahbour L, Gibbs J, Coletta C, Hummell J, Al-Sarie M (2020). Peritoneal dialysis zoonotic bacterial peritonitis with *Staphylococcus pseudintermedius*. Case Rep Nephrol Dial.

[44] Nomoto H, Kutsuna S, Nakamura K, Nakamoto T, Shimomura A (2020). Totally implantable venous access port infection caused by *Staphylococcus pseudintermedius*: possible transmission from a companion dog to a human. J Infect Chemother.

[45] Kitagawa H, Hisatsune J, Ohge H, Kutsuno S, Hara T (2021). Implanted port catheter system infection caused by methicillin-resistant *Staphylococcus pseudintermedius* ST71-SCCmec type III. Intern Med.

[46] Chuang C, Yang Y, Hsueh P, Lee P (2010). Catheter-related bacteremia caused by *Staphylococcus pseudintermedius* refractory to antibiotic-lock therapy in a hemophilic child with dog exposure. J Clin Microbiol.

[47] Diaz MA, Gardner LB, Libertin CR (2019). *Staphylococcus pseudintermedius* catheter-related bloodstream infection after exposure to domestic dogs and a cat. *BMJ Case Rep*.

[48] Östholm Balkhed Å, Söderlund R, Gunnarsson L, Wikström C, Ljung H (2023). An investigation of household dogs as the source in a case of human bacteraemia caused by *Staphylococcus pseudintermedius*. Infect Ecol Epidemiol.

[49] Subedi P, Syed MP, Kate Y, Koirala B (2021). *Staphylococcus pseudintermedius*: a common zoonotic pathogen causing postprocedural urosepsis in humans. BMJ Case Rep.

[50] Van Hoovels L, Vankeerberghen A, Boel A, Van Vaerenbergh K, De Beenhouwer H (2006). First case of *Staphylococcus pseudintermedius* infection in a human. J Clin Microbiol.

[51] Riegel P, Jesel-Morel L, Laventie B, Boisset S, Vandenesch F (2011). Coagulase-positive *Staphylococcus pseudintermedius* from animals causing human endocarditis. Int J Med Microbiol.

